# Response study of tower crane and elastic plate under dynamic complicated excitation

**DOI:** 10.1371/journal.pone.0324745

**Published:** 2025-06-27

**Authors:** Fu Liu, Haopeng Chen

**Affiliations:** School of Mechanical and Electrical Engineering, Beijing Institute of Graphic Communication, Beijing, People’s Republic of China; Tecnológico de Monterrey, MEXICO

## Abstract

Structural vibration will inevitably occur under the dynamic complicated excitations in tower crane and elastic plate system. The continuous nonlinear vibration would lead to the deterioration of the coupling state of the responses of the tower crane and the elastic plate system. This, in turn, impacts the operational stability and reduces the service life of components. Consequently, there has been scant research on response calculation of the structure in a tower crane and elastic plate system when considering such dynamic and complex excitations. To address this gap, this paper employs a spatially coupled dynamics model of the tower crane and the plate, covering the acceleration stage, the constant speed stage, and then the deceleration stage. This model incorporates typical air resistance, the Lagrange equation, and the calculation equations based on the Reissner plate theory. Additionally, emergency braking, regarded as a type of fault, is taken into account in the dynamic model. The present model is solved numerically. The feasibility of the model is validated by comparing the measured responses of the dynamic coupling model with the experimental ones. An extensive analysis is conducted on the influence of different braking times on the dynamic response. The analysis reveals that if the emergency braking time exceeds 1.5 seconds, the vibration of the plate and tower crane system is minimal. The Kurtosis Value, Factor Value, and Skewness Value exhibit high sensitivity to the dynamic responses of the plate and the swing angles. The Frequency Center demonstrates high sensitivity to the swing angle, while the Root Mean Square Frequency and Frequency Standard Deviation show high sensitivity to the plate vibration.

## Introduction

The tower crane serves as a typical illustration of a steel structure. In engineering construction, safety accidents in the coupling system of the tower crane and foundation plate have happened frequently, giving rise to economic losses as well as casualties [1].

The dynamic performance analysis of crane systems has been studied extensively and deeply by many scholars. Crane model was degenerated to consider two classical cases-the moving mass case and the moving payload case [2]. Lifting mechanism and span structure of the nuclear power crane were needed to perform predominantly in serious working conditions [3]. Coupling system of an overhead crane was simplified to that of a moving mass with pendulum swing passing beam model [4]. Constrained dynamics was derived using an automated computational procedure dedicated to constrained systems. The procedure was implemented to rigid system models [5]. The tower crane was simplified as a payload-trolley-jib system [6]. Tower crane and the hoisting rope were considered flexible. The rigid flexible coupling model of crane system was established by Lagrangian equation [7]. The four degrees-of-freedom nonlinear mathematical model was developed. The model represented the seismic behavior of gantry cranes [8]. A bolt-connected prefabricated cross-shaped I-steel tower crane foundation was proposed [9]. Tower crane mast was considered and aimed to obtain a scaled physical model. The model dynamically represented the real structure in order to experimentally investigate [10]. The differential equations of tower vibration, jib vibration, and payload swing were derived under the radial motion by the Lagrangian equation. The vibration modes of the tower were analyzed [11]. The moving load model was established. The transient dynamic analysis method was used to carry out dynamic simulation, analyze and obtain the vibration characteristics of the boom under luffing and lifting [12]. The wind tunnel model was used for the simulation of tower cranes under full wind angles and analyzed the aerodynamic load variation of the tower crane under full wind angles [13]. The stochastic response analysis and reliability assessment of wind-induced vibration of the crane were addressed with novel simulation method for stochastic wind fields [14]. The influence degree of five variable parameters of clamping bushing on the bearing capacity of the structure was investigated [15]. By obtaining the stress ratio and maximum deformation before and after integrated optimization design, a local strengthening scheme for the tower crane support tube was proposed to simplify the structural connection [16]. The electromechanical rigid-flexible coupling were established model for tower cranes to simulate the characteristics of load swing caused by flexible transmission and electromechanical nonlinear coupling [17]. The dynamics model of the coupled jib-cargo vibration system of a flat-top tower crane under the luffing working condition was established. The influence law of system parameters on cargo swing was analyzed [18], without describing foundation plate analysis and the effects of coupling motions.

The impact of plate has not been considered on the complicated dynamic characteristics. Composite foundation constitutive model was proposed to determine the equivalent elastic modulus of the concrete foundation [19]. Foundation of the plate is modelled as a fractionally-damped Kelvin-Voigt model [20]. The governing equation of the functionally graded plate was formulated in a coordinate system which moved along with the applied load [21]. The parametric earthquake analysis of thick plates resting on Winkler foundation was studied [22]. Responses of functionally graded plate resting on a Winkler-Pasternak elastic foundation was studied [23]. The high-order shear deformation theory was introduced to compute the free vibration of the cracked functionally graded material plates according to the power law resting on Pasternak elastic foundations [24]. The vibration of a moderately thick plate to a moving mass was investigated. An analytical-numerical solution was proposed based on the eigenfunction expansion method [25]. The free vibration analysis of an axially traveling moderately thick FG plate was carried out using the finite element method [26]. Benchmark analytic solutions were studied for the free vibration of L-shaped moderately thick plates [27]. The mathematical model was presented to address the nonlinear buckling, postbuckling, and snap-through of bidirectional functionally graded porous simply supported plates rested on elastic foundation and subjected to uniaxial/biaxial compressive loads [28]. The simple refined shear deformation theory that eliminated the use of a shear correction factor was presented for buckling analysis of in-plane bi-directional functionally graded porous plates [29]. General visco-Winkler-Pasternak foundation was created to analyze the vibrations of functionally graded sandwich plates [30]. The governing equations were derived, which were solved utilizing Navier’s technique to determine the deflection of a simply supported functionally graded graphene platelet-reinforced ceramic-metal plate [31]. The governing equations of motion were derived using a simple higher-order shear deformation theory and Hamilton’s principle [32].

The research on the response study of the structure in the crane tower – elastic foundation plate system has been extensive and elaborate. Nevertheless, it is typically carried out on the pure crane tower and elastic plate, without taking into account the complex external excitation. In this paper, theoretical model section presents the model of the crane tower and elastic plate system. In model verification section, the experimental design is introduced, which puts forward a refined coupling motion model and makes comparisons with the experimental results. Motion response and analysis section elaborates on the structural dynamic response and indicator characteristics of the model under the complex external excitation. Conclusions are drawn in final section.

## Theoretical model

**Fig 1:**
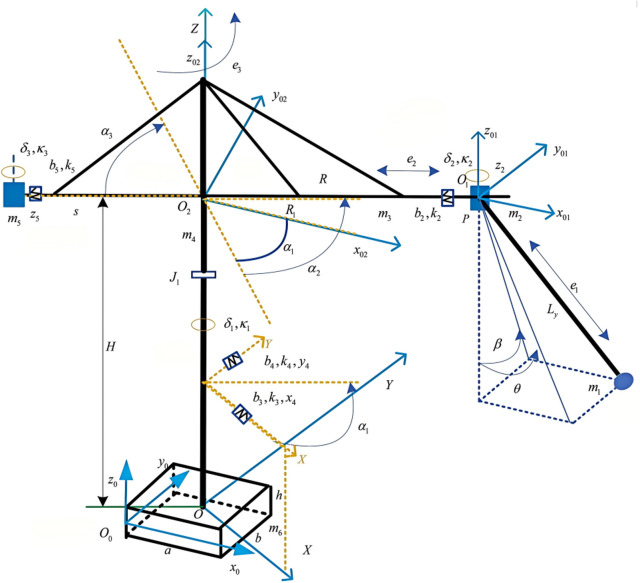
Schematic of the crane tower-plate spatially coupled dynamics model.

In order to obtain the dynamic state parameters of the response during the complex operation in the tower crane and elastic plate system, this paper employs a spatially coupled dynamics model of the crane tower and elastic plate system under emergency braking and coupling motion conditions. The schematic diagram of the crane tower and plate coupled dynamics model is illustrated in Fig 1.

### Tower crane system model

The tower crane system is conceptualized as a multi-rigid body system, which consists of the mast, boom, trolley, rope, counterweight, and payload. The mass and moment of inertia of each component are concentrated at their corresponding mass centers. A spring-damping vibration model is employed for each support element. The independent generalized coordinates among the mast, boom, trolley, rope, counterweight, and payload can be ascertained as


$$\left[\begin{array}{c} X_{1}\\ Y_{1}\\ Z_{1}\\ X_{3}\\ Y_{3}\\ Z_{3}\\ X_{4}\\ Y_{4}\\ Z_{4}\\ X_{5}\\ Y_{5}\\ Z_{5}\\ X_{6}\\ Y_{6}\\ Z_{6} \end{array}\right]=[\begin{array}{ccccccccccccccc} \cos\alpha_{5} & -\sin\alpha_{5} & 0 & \cos\alpha_{5} & 0 & 0 & \cos\alpha_{1} & -\sin\alpha_{1} & 0 & 0 & 0 & 0 & 1 & 0 & 0\\ \text{sin}\alpha_{5}\text{cos}\delta & \text{cos}\alpha_{5}\text{cos}\delta & -\text{sin}\delta & \text{sin}\alpha_{5}\text{cos}\delta & 0 & \text{sin}\delta & \text{sin}\alpha_{1}\text{cos}\delta +2R(\text{sin}\delta )/H & \text{cos}\alpha_{1}\text{cos}\delta & \text{sin}\delta & 0 & 0 & 0 & 0 & \text{cos}\delta & \text{sin}\delta \\ -\sin\alpha_{2}\sin\delta & \cos\alpha_{2}\cos\delta & -\cos\delta & -\sin\alpha_{5}\sin\delta & 0 & \cos\delta & 2R(\cos\delta )/H-\sin\alpha_{1}\sin\delta & -\sin\delta \cos\alpha_{1} & \cos\delta & 0 & 0 & 0 & 0 & \text{-sin}\delta & \cos\delta \\ \begin{array}{c} \begin{array}{c} \begin{array}{c} \begin{array}{c} 0 \end{array} \end{array} \end{array} \end{array} & 0 & 0 & \cos\alpha_{5} & 0 & 0 & \cos\alpha_{1} & -\sin\alpha_{1} & 0 & 0 & 0 & 0 & 1 & 0 & 0\\ 0 & 0 & 0 & \sin\alpha_{5}\text{cos}\delta & 0 & \text{sin}\delta & \text{sin}\alpha_{1}\text{cos}\delta +2R(\text{sin}\delta )/H & \text{cos}\alpha_{1}\text{cos}\delta & \text{sin}\delta & 0 & 0 & 0 & 0 & \text{cos}\delta & \text{sin}\delta \\ 0 & 0 & 0 & -\sin\alpha_{5}\sin\delta & 0 & \cos\delta & 2R(\cos\delta )/H-\sin\alpha_{1}\sin\delta & -\sin\delta \cos\alpha_{1} & \cos\delta & 0 & 0 & 0 & 0 & -\sin\delta \text{\textbackslash{}quad} & \cos\delta \\ 0 & 0 & 0 & 0 & 0 & 0 & \cos\alpha_{1} & -\sin\alpha_{1} & 0 & 0 & 0 & 0 & 1 & 0 & 0\\ 0 & 0 & 0 & 0 & 0 & 0 & \sin\alpha_{1}\cos\delta & \cos\alpha_{1}\cos\delta & \sin\delta & 0 & 0 & 0 & 0 & \cos\delta & \sin\delta \\ \text{\textbackslash{}quad}0\text{\textbackslash{}quad} & 0 & 0 & 0 & 0 & 0 & -\sin\delta \sin\alpha_{1} & -\sin\delta \cos\alpha_{1} & \cos\delta & 0 & 0 & 0 & 0 & -\sin\delta & \cos\delta \\ 0 & 0 & 0 & 0 & 0 & 0 & -\sin\alpha_{1} & -\sin\alpha_{1} & 0 & \cos\alpha_{6} & 0 & 0 & 1 & 0 & 0\\ 0 & 0 & 0 & 0 & 0 & 0 & \sin\alpha_{1}\cos\delta +2s(\sin\delta )/H & \cos\alpha_{1}\cos\delta & \sin\delta & \sin\alpha_{6}\cos\delta & 0 & \sin\delta & 0 & \cos\delta & \sin\delta \\ 0 & 0 & 0 & 0 & 0 & 0 & 2s(\cos\delta )/H-\sin\alpha_{1}\sin\delta & -\sin\delta \cos\alpha_{1} & \cos\delta & -\sin\delta \sin\alpha_{6} & 0 & \cos\delta & 0 & -\sin\delta & \cos\delta \\ 0 & 0 & 0 & 0 & 0 & 0 & 0 & 0 & 0 & 0 & 0 & 0 & 1 & 0 & 0\\ 0 & 0 & 0 & 0 & 0 & 0 & 0 & 0 & 0 & 0 & 0 & 0 & 0 & \cos\delta & \sin\delta \\ 0 & 0 & 0 & 0 & 0 & 0 & 0 & 0 & 0 & 0 & 0 & 0 & 0 & -\sin\delta & \cos\delta \end{array}]\left[\begin{array}{c} x_{1}\\ y_{1}\\ z_{1}\\ x_{3}\\ y_{3}\\ z_{3}\\ x_{4}\\ y_{4}\\ z_{4}\\ x_{5}\\ y_{5}\\ z_{5}\\ x_{6}\\ y_{6}\\ z_{6} \end{array}\right]$$ (1)

Where the axis *x*_1_ and *z* are parallel to the axis *X* and *Z* of the coordinate *XYZ* system. Given the small mass of the trolley, *X*_2_, *Y*_2_, and *Z*_2_ have been disregarded in the solution.


$$\nu_{p}^{2}={\dot{X}}_{p}^{2}+{\dot{Y}}_{p}^{2}+{\dot{Z}}_{p}^{2},p=1.3,4,5,6$$ (2)

$$T=\frac{1}{2}\sum_{p=1}^{6}\left(J_{p}{\dot{\alpha }}_{p}^{2}+m_{p}\nu_{p}^{2}\right)$$ (3)

$$V=\sum_{p=1}^{6}\left(\frac{1}{2}k_{p}s_{p}^{2}+\frac{1}{2}\kappa_{p}\gamma_{p}^{2}+m_{p}gh_{p}\right)$$ (4)

Where the *X*_*p*_, *Y*_*p*_ and *Z*_*p*_ are the *p*’th mass displacements.

Swing displacements and rope length of payload are expressed as


$$x_{1}=L_{y}\sin\theta$$ (5)

$$y_{1}=L_{y}sin\beta$$ (6)

$$Q_{i}=Q_{r,pi}+Q_{d,i}$$ (7)

Where $Q_{r,pi}$ and $Q_{d,i}$ respectively represent the air resistance and the damping. The damping and air resistance are described as


$$Q_{r,pi}=-\frac{1}{2}c\rho_{p}\nu_{p}^{2}S_{p}$$ (8)

$$Q_{d,i}=-d_{i}{\dot{q}}_{i}$$ (9)

Where the symbols *ρ*_*p*_, *c*, *S*_*p*_ and *d*_*i*_ stand for air density, air resistance coefficient, windward area and damping coefficient, respectively.

### Boom deflection model

The boom deflection deformation is expressed as


w=∑∫Mκds (10)

$$\begin{array}{c} F=m_{1}\left(e_{1}+g\left(\cos\theta +\cos\beta \right)+L_{y}\left({\dot{\theta }}^{2}+{\dot{\beta }}^{2}\right)\right)\left(\cos\theta +\cos\beta \right)\\ +m_{1}e_{3}\dot{\theta }\sin\beta \left(L_{y}\sin\theta +2t\cos\theta \right)\left(\cos\theta +\cos\beta \right) \end{array}$$ (11)

Where the *w*, *M*, *F*, *ds* and *κ* are the boom deflection, stress resultant force on both sides of the micro-segment, the micro-segment, the concentrated force and micro-segment bending deformation, respectively.

The angles *δ*_5_ and *δ*_6_ represent the rotation of the boom and the rotation of the girder around the mast, respectively. The rope length *L*_*y*_, the *δ*_5_ angle and the *δ*_6_ angle are expressed as


$$\begin{array}{c} L_{y}=\left\{ \begin{array}{c} @cL-e_{1}t^{2}/2,0⩽t⩽t_{1}\\ L-3e_{1}t_{1}^{2}/2-e_{1}t_{1}t,t_{1}⩽t⩽t_{2}\\ L-3e_{1}t_{1}^{2}/2-et_{1}t+e_{1}{\left(t-t_{1}\right)}^{2}/2,t_{2}⩽t⩽t_{3} \end{array}\right.\; \end{array}$$ (12)

$$\begin{array}{c} R_{2}=\left\{ \begin{array}{c} @cR_{1}-\frac{1}{2}e_{2}t^{2},0⩽t⩽t_{1}\\ R_{1}-\frac{3}{2}e_{2}t_{1}^{2}-e_{2}t_{1}t,t_{1}⩽t⩽t_{2}\\ R_{1}-\frac{3}{2}e_{2}t_{1}^{2}-e_{2}t_{1}t+\frac{1}{2}e_{2}{\left(t-t_{2}\right)}^{2},t_{2}⩽t⩽t_{3} \end{array}\right.\; \end{array}$$ (13)

$$\begin{array}{c} \;\delta_{4}=\left\{ \begin{array}{c} @l\delta_{2}+e_{3}t^{2}/r,0⩽t⩽t_{1}\\ \delta_{2}+e_{3}t_{1}t/r,t_{1}⩽t⩽t_{2}\\ \delta_{2}+e_{3}(4t_{1}t_{2}+2t_{1}t+{\left(t-t_{2}\right)}^{2})/2r,t_{2}⩽t⩽t_{3} \end{array}\right.\; \end{array}$$ (14)

$$\delta_{5}=\delta_{4}+\pi +\delta_{3}-\delta_{2}$$ (15)

Where the *t*_*1*_, *t*_*2*_, *t*_*3*_, *R*_*1*_ and *r* respectively represent accelerating maximum time, constant speed maximum time, decelerating maximum time, initial boom length, and gear radius of slewing motor.

### Lagrange equation

The equations (1)–(15) are substituted into equation (16) to yield the following motion matrix.


$$\frac{d}{dt}\left(\frac{\partial T}{\partial {\dot{q}}_{i}}\right)-\frac{\partial T}{\partial q_{i}}+\frac{\partial V}{\partial q_{i}}=Q_{i}\;(i=1,\cdots ,i)$$ (16)

$$\;[\begin{array}{ccc} B_{0101} & \cdots & B_{0115}\\ \cdots & \ddots & \cdots \\ B_{1501} & \cdots & B_{1515} \end{array}]\left[\begin{array}{c} {\ddot{q}}_{1}\\ {\ddot{q}}_{2}\\ {\ddot{q}}_{3}\\ {\ddot{q}}_{4}\\ {\ddot{q}}_{5}\\ {\ddot{q}}_{6}\\ {\ddot{q}}_{7}\\ {\ddot{q}}_{8}\\ {\ddot{q}}_{9}\\ {\ddot{q}}_{10}\\ {\ddot{q}}_{11}\\ {\ddot{q}}_{12}\\ {\ddot{q}}_{13}\\ {\ddot{q}}_{14}\\ {\ddot{q}}_{15} \end{array}\right]=\left[\begin{array}{c} D_{1}\\ D_{2}\\ D_{3}\\ D_{4}\\ D_{5}\\ D_{6}\\ D_{7}\\ D_{8}\\ D_{9}\\ D_{10}\\ D_{11}\\ D_{12}\\ D_{13}\\ D_{14}\\ D_{15} \end{array}\right]$$ (17)

The equation (17) represents thirteen second-order non-linear differential equations with non-constant coefficients. It is worth noting that the mast interaction is the medium of coupling between payload and foundation plate.

### Dynamic equations of the foundation plate system

The relations between stress and internal force of plate can be defined as


$$\left[\begin{array}{c} {}*20cM_{xx}\\ M_{yy}\\ M_{xy}\\ M_{yx}\\ Q_{x}\\ Q_{y} \end{array}\right]=\int_{-h}^{\frac{h}{2}}\left[\begin{array}{c} {}*20cz\sigma_{xx}\\ z\sigma_{yy}\\ z\tau_{xy}\\ z\tau_{yx}\\ \tau_{xz}\\ \tau_{yz} \end{array}\right]dz$$ (18)

Where the symbols *M*_*xx*_, *M*_*yy*_, *M*_*yx*_, σ_*xx*_, σ_*yy*_ and σ_*xy*_ respectively stand for the internal force and stress.

The vibration displacement of crane plate is *w*_11_. The vibration displacement is *w*_1_ after the crane plate settles at certain angle *δ*.


$$w_{1}=w_{11}\cos\delta$$ (19)

Based on Reissner mid-plane plate theory and elasticity theory, The relationship between stress, internal force and external force can be derived as


$$\begin{array}{c} \sigma_{xx}=\frac{12M_{xx}}{h_{1}^{3}}z_{6},\sigma_{yy}=\frac{12M_{yy}}{h_{1}^{3}}z,\sigma_{zz}=-2q{\left(\frac{1}{2}-\frac{z}{h_{1}}\right)}^{2}\left(1+\frac{z}{h_{1}}\right)\\ \tau_{xz}=\frac{3(h_{1}^{2}-4z^{2})Q_{x}}{2h_{1}^{3}},\tau_{yz}=\frac{3(h_{1}^{2}-4z^{2})Q_{y}}{2h_{1}^{3}},\tau_{xy}=\frac{12M_{xy}}{h_{1}^{3}}z_{6} \end{array}$$ (20)

Where the *Q*_*x*_, *Q*_*y*_, *τ*_*xz*_, *τ*_*yz*_ and *τ*_*xy*_ are the internal force and shear stress of the mid-plane plate, respectively. Based on equilibrium for plate, the internal force and external force can be expressed as


$$Q_{x}=\frac{\partial M_{xx}}{\partial x}+\frac{\partial M_{yx}}{\partial y}$$ (21)

$$Q_{y}=\frac{\partial M_{yy}}{\partial x}+\frac{\partial M_{xy}}{\partial y}$$ (22)

$$q(x,y)=\frac{\partial Q_{x}}{\partial x}-\frac{\partial Q_{y}}{\partial y}$$ (23)

$$\left[\begin{array}{c} {}*20cM_{xx}\\ M_{yy}\\ M_{xy} \end{array}\right]=D\left[\begin{array}{c} {}*20c\frac{\partial^{2}w_{11}}{\partial x_{6}^{2}}+\mu \frac{\partial^{2}w_{11}}{\partial y_{6}^{2}}\\ \frac{\partial^{2}w_{11}}{\partial y_{6}^{2}}+\mu \frac{\partial^{2}w_{11}}{\partial x_{6}^{2}}\\ (1-\mu )\frac{\partial^{2}w_{11}}{\partial y_{6}\partial x_{6}} \end{array}\right]+\frac{h_{1}^{2}}{10}\left[\begin{array}{c} {}*20c2\frac{\partial Q_{x}}{\partial x_{6}}-\frac{\mu q}{1-\mu }\\ 2\frac{\partial Q_{y}}{\partial y_{6}}-\frac{\mu q}{1-\mu }\\ \frac{\partial Q_{x}}{\partial y_{6}}+\frac{\partial Q_{y}}{\partial x_{6}} \end{array}\right]$$ (24)

Where the symbols *D* and *µ* are the bending stiffness and Poisson’s ratio of the mid-plane plate, respectively. Based on equilibrium for plate, the average deflection *w*11 can be derived as


$$D\nabla^{4}w_{11}=q-\frac{(2-\mu )h_{1}^{2}}{10\left(1-\mu \right)}\nabla^{2}q$$ (25)

in which $\nabla^{2}=\frac{\delta^{2}}{\delta x_{6}^{2}}+\frac{\delta^{2}}{\delta y_{6}^{2}},\nabla^{4}=\frac{\delta^{4}}{\delta x_{6}^{4}}+\frac{\delta^{4}}{\delta x_{6}^{2}\delta y_{6}^{2}}+{\frac{\delta^{4}}{\delta y_{6}^{4}}}_{\leftarrow }$

Utilizing whole motion equations, the vibration response of the structures are calculated.

When the crane foundation system undergoes emergency braking under coupling conditions, the deceleration of the lifting mechanism, the slewing mechanism, and the luffing mechanism changes instantaneously. Owing to the inertia of the system, the structural parameters stay in their original states. Consequently, all the structural dynamic responses of the system also remain unchanged for a brief period. The mapping relationship between time and the various deceleration has been established as


$$\begin{array}{c} \;0=\left\{ \begin{array}{c} @le_{1}t-e_{11}t_{11},0⩽t⩽t_{1}\\ e_{1}t_{1}-e_{11}t_{12},t_{1}⩽t⩽t_{2}\\ e_{1}\left(t_{1}+t_{2}-t_{3}\right)-e_{11}t_{13},t_{2}⩽t⩽t_{3} \end{array}\right.\; \end{array}$$ (26)

$$\begin{array}{c} \;0=\left\{ \begin{array}{c} @le_{2}t-e_{21}t_{21},0⩽t⩽t_{1}\\ e_{2}t_{1}-e_{21}t_{22},t_{1}⩽t⩽t_{2}\\ e_{2}(t_{1}+t_{2}-t_{3})-e_{21}t_{23},t_{2}⩽t⩽t_{3} \end{array}\right.\; \end{array}$$ (27)

$$\begin{array}{c} \;0=\left\{ \begin{array}{c} @le_{3}t/r-e_{31}t_{31}/r,0⩽t⩽t_{1}\\ e_{3}t_{1}/r-e_{31}t_{32}/r,t_{1}⩽t⩽t_{2}\\ e_{3}(t_{1}+t_{2}-t_{3})/r-e_{31}t_{33}/r,t_{2}⩽t⩽t_{3} \end{array}\right.\; \end{array}$$ (28)

Where the $\;e_{11}$, $e_{21}$ and $e_{31}$ respectively represent the lifting deceleration, luffing deceleration, and slewing deceleration for emergency braking. The *t*_11_, *t*_12_ and *t*_13_ are the emergency braking duration during the acceleration phase, constant speed phase, and deceleration phase of the lifting mo*t*ion, respec*t*ively. The *t*_21_, *t*_22_ and *t*_31_ are the emergency braking duration during the acceleration phase, constant speed phase, and deceleration phase of the luffing motion, respec*t*ively. The *t*_31_, *t*_32_ and *t*_33_ are the emergency braking duration during the acceleration phase, constant speed phase, and deceleration phase of the slewing motion, respec*t*ively.

In conjunction with the coupling system model of the tower crane foundation plate, the dynamic responses of the foundation plate and the tower crane at various time points during emergency braking can be computed by employing the simultaneous equations (26)–(28).

## Model verification

**Fig 2:**
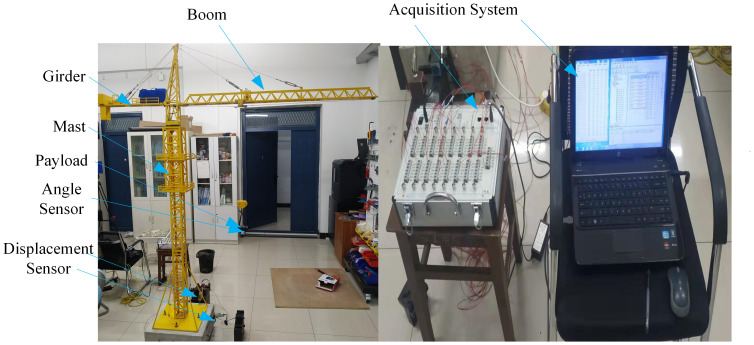
Components of the test bench.

The scale ratio of the tower crane-base plate system model can be deduced using the Buckingham pi theorem [10,33]. The test bench was designed as a scaled-down tower crane to test, verify, and collect data in Fig 2. Each system operation consists of three stages: acceleration, constant speed, and deceleration. The lifting acceleration, slewing acceleration, and luffing acceleration were respectively 0.003 m/s², 0.02 m/s², and 0.001 m/s². During testing time, three different crane loads were selected for experimentation to ensure the safety of the tower crane-foundation plate dynamic experiments. The masses of payloads were 2.5 kg, 5 kg, and 10 kg, with each condition repeated three times. Based on readings displayed by the angle sensor on the server, the initial swing angles of the payloads were fine-tuned. The operation time was divided into three phases: from 0 s to 3 s for acceleration, from 3 s to 15 s for constant speed, and from 15 s to 18 s for deceleration. The experiment was conducted within the structural laboratory. The experimental procedure was executed as detailed below. Firstly, the scaled down tower crane was installed on the test platform. Secondly, angle sensors, strain gauges, displacement meters, and the equipment of the acquisition system were mounted. Thirdly, payloads were loaded onto the hook of the operating tower crane incrementally until the normal working loading condition was achieved. Then, to realistically simulate the motion behaviors, the structural dynamics of the crane were considered. Each parameter of the tower crane was tested during the lifting and slewing motions, with the motion being precisely time-controlled. Finally, all measured parameters were systematically recorded step by step by the computer through the data acquisition system equipment. The layout of the integrated test setup is presented in Fig 2.

The design parameter values of plate and tower crane are provided in Table 1.

**Table 1:** Parameter values of the tower crane-plate.

Parameters	values	Parameters	values
Lifting acceleration *e*_1_ (m/s^2^)	3e-3	Mast mass *m*_4_ (kg)	55
Slewing acceleration *e*_3_ (m/s^2^)	0.02	Counterweight mass *m*_5_ (kg)	15
Boom bending stiffness *D* (N/m)	3914	Boom mass *m*_3_ (kg)	10
Air resistance coefficient *c*	0.2	Rope length *L* (m)	2
Moment of inertia *J*_1_ (m^4^)	7.375e-3	Boom length *R*_1_ (m)	1.5
Mast length *H* (m)	2	Gravitational acceleration *g* (m/s^2^)	9.8
Girder length *s* (m)	0.5	Plate thickness *h*_1_ (m)	0.2
Plate elastic modulus *E*_*c*_ (MPa)	2.9e4	Plate length *a* (m)	0.5
Poisson’s ratio *µ*	0.167	Plate width *b* (m)	0.5

**Fig 3:**
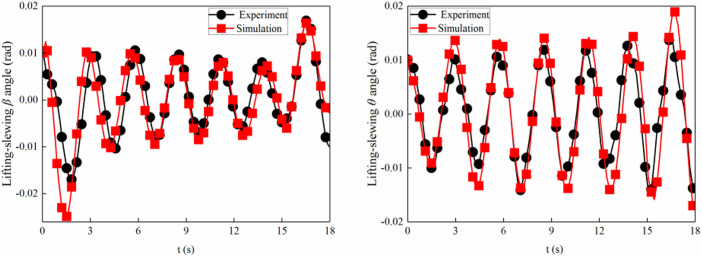
The swing angles of the experiment and simulation.

The initial angles of the experiment and simulation of the crane foundation system are 0.01 rad. In the experiment, the tower crane and foundation plate system simultaneously perform lifting and slewing motion, and the excitation for the movements is provided by lifting accelerations and slewing accelerations. The validation model applies the comparison of swing angle *β* in Fig 3(a) and swing angle *θ* in Fig 3(b) between the experiment and simulation results under the lifting and slewing motion. The change trends of simulation and experiments are highly similar.

## Motion response and analysis

When an unforeseen scenario emerges, the tower crane and foundation plate coupling system is compelled to perform emergency braking under coupled circumstances. Owing to the action of inertia, the structural components of the tower crane and foundation plate are likely to sustain vibrations, and the system has a propensity to experience pronounced oscillations. Analyzing the emergency braking response characteristics of the tower crane and foundation plate system from a dynamic vantage point is instrumental in safeguarding the secure and stable operation of this coupling system.

### Impact of emergency braking on the dynamic response

**Fig 4:**
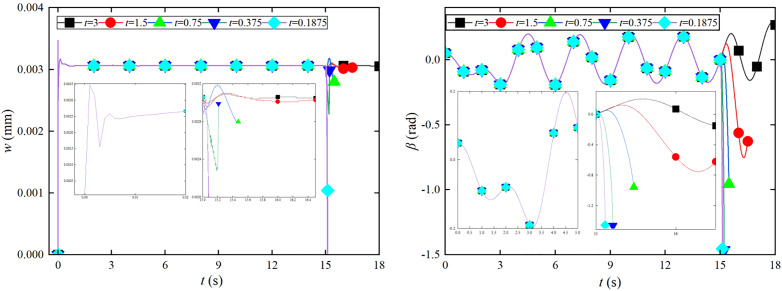
Effect of the different braking time on the foundation plate and *β* angle. (a) *w* response of the lifting and slewing motion. (b) *β* response of the lifting and slewing motion.

Emergency braking analysis is conducted on the tower crane and foundation plate coupling system under the coupling lifting and slewing motion. Further dynamic characteristics analysis is conducted on vibration of the foundation plate and the swing angle vibration of the lifting load using different braking times, as shown in Fig 4.

The impact on the amplitude of the foundation plate *w* increases in Fig 4(a). When the braking time is not less than 1.5s, the impact of emergency braking on the vibration of foundation plate is relatively small. As the braking time decreases exponentially, emergency braking causes vibration of the foundation plate to increase exponentially. When the braking time is not greater than 0.75 s, the vibration of the foundation plate increases sharply. When the emergency braking time is not greater than 0.1875 s, it causes serious damage to the foundation plate. As the emergency braking time increases in Fig 4(b), the angular amplitude of *y*-direction *β* significantly increases. The period of angle *β* vibration increases. When the emergency braking time is not less than 3s, the influence of angular *β* amplitude is relatively small. When the emergency braking time is not more than 0.375s, the sharp increase in angular *β* amplitude causes severe vibration damage to the tower crane structure. The emergency braking time is 0.75 s, the maximum amplitude of the angle *β* can reach −0.9407 rad. Emergency braking causes a larger swing angle. The braking time is shortened to 1.5 s, and the maximum amplitude of the angle *β* can reach −0.7545 rad.

**Fig 5:**
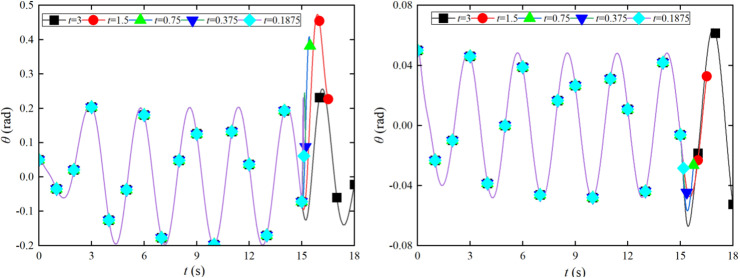
Effect of the different braking time on the *θ* angles. (a) *θ* response of lifting and slewing motion. (b) *θ* response of luffing and slewing motion.

The emergency braking has an impact on the angular *θ* amplitude under the condition of lifting and slewing motion when the emergency braking time is from 0.375 s to 1.5 s in Fig 5(a). As the emergency braking time shortens, there is a decreasing trend in the amplitude of angular *θ* vibration. The reason for the shrinkage may be that the initial angle position does not match the slewing direction. When the emergency braking time is less than 1.5 s, the angular *θ* amplitude shows a decreasing trend. As the emergency braking time shortens under the condition of coupled luffing and slewing motion, the angular *θ* amplitude decreases continuously in Fig 5(b). The angle *θ* shows a divergent trend during the deceleration phase. Reducing the emergency braking time is benefit to divergence trend of the angle *θ*. The impact of emergency braking time shows a decreasing trend when the emergency braking time is not greater than 0.375 s.

**Fig 6:**
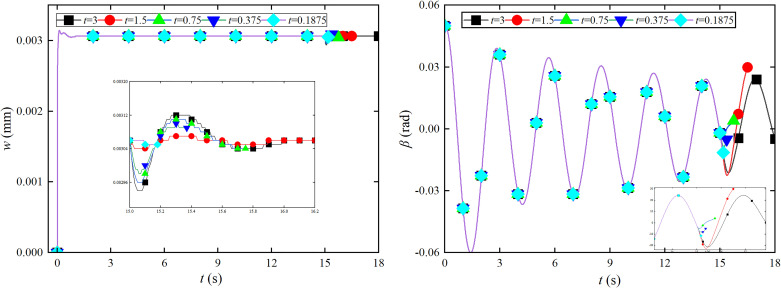
Effect of the different braking time on the foundation plate and *β* angle. (a) *w* response under the luffing and slewing motion. (b) *β* response under the luffing and slewing motion.

As the emergency braking time decreases when the emergency braking time is not greater than 0.75 s under the luffing and slewing motion, the *w* value of the foundation plate shows a decreasing trend in Fig 6(a). The impact of emergency braking on the displacement of the foundation plate is minimal when the emergency braking time is 0.187 s. The impact of the emergency braking time on the displacement amplitude is the greatest when the emergency braking time is 3 s. Emergency braking reduces the amplitude of the foundation plate displacement during the deceleration phase. The displacement amplitude of the foundation plate is maximum during the deceleration stage when the emergency braking time is 1.5 s in Fig 6(b). When the emergency braking time is not greater than 0.75 s, the displacement amplitude of the foundation plate also decreases as the braking time decreases.

### Impact of emergency braking on the structural indicator

The impact of different emergency braking times is analyzed in the displacement of foundation plate and the vibration of tower crane structure through seven time-domain indicators and three frequency-domain indicators. The time-domain indicators contain Peak to Peak Value (PPV), Square Root of the Amplitude Value (SRA), Pulse Factor Impulse Factor Value (IF), Root Mean Square Value (RMS), Kurtosis Value (KV), Peak Value (PV), and Skewness Values (SV) in Table 2. The Frequency-Domain Indicators Contain the Frequency Center (FC), Root Mean Square Frequency (RMSF) and Root Variance Frequency (RVF) in Table 3.

**Table 2:** Expression of time domain indicators.

Abbreviation	Mathematical expression
PPV	$X_{PPV}=\max(x_{i})-\min(x_{i})$
SRA	$X_{SRA}={\left(\frac{1}{N}\sum_{i=1}^{N}\sqrt{\left|x_{i}\right|}\right)}^{2}$
IF	$X_{IF}=\left(\max|x_{i}|\right)/\left(\frac{1}{N}\sum_{i=1}^{N}|x_{i}|\right)$
RMS	$X_{RMS}=\sqrt{\frac{1}{N}\sum_{i=1}^{N}x_{i}^{2}}$
KV	$X_{KV}=\frac{1}{N}\sum_{i=1}^{N}{\left(\frac{x_{i}-\bar{x}}{\sigma }\right)}^{4}$
SV	$X_{SV}=\frac{1}{N}\sum_{i=1}^{N}{\left(\frac{x_{i}-\bar{x}}{\sigma }\right)}^{3}$
PV	$X_{PV}=\frac{1}{N}\sum_{i=1}^{N}x_{i}$

**Table 3:** Expression of time domain indicators.

Abbreviation	Mathematical expression
FC	$X_{FC}=\frac{1}{N}\sum_{i=1}^{N}f_{i}$
RMSF	$X_{RMSF}=\sqrt{\frac{1}{N}\sum_{i=1}^{N}f_{i}^{2}}$
RVF	$X_{RVF}=\sqrt{\frac{1}{N}\sum_{i=1}^{N}{\left(f_{i}-X_{FC}\right)}^{2}}$

The response characteristics of the tower structure and foundation plate are studied by changing different emergency braking times. The braking time levels are divided into four levels, corresponding to 1.5 s, 0.75 s, 0.375 s, and 0.1875 s, respectively. The structural indicator changes of the tower crane foundation plate coupling system with respect to the braking time of 3 s are studied.

**Fig 7:**
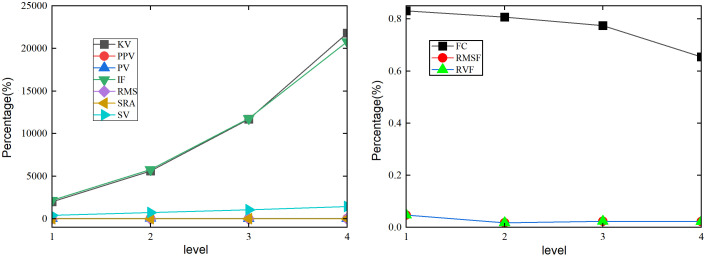
Effect of the different braking time on the angle under the lifting and slewing coupling motion. (a) Time domain indicators of *θ.* (b) Frequency domain indicators of *θ*.

As the braking time of the tower crane foundation plate coupling system coupled with lifting and slewing motion decreases in Fig 7(a), the sensitivity of the time-domain indicator SRA is poor in changes in the healthy state of the angle *θ*. The sensitivity of the time-domain indicators KV, PPV, IF, RMS, PV, and SV are high to changes in the healthy state of the swing angle *θ*. As the braking time level increases, the health status changes of KV, IF, RMS, and SV show monotonicity on the suspension swing angle *θ*. PPV and PV have a consistent trend in the health status changes of swing angle *θ*. When the braking time level is 4, the response of KV, IF, and SV to the state change of the angle *θ* shows the maximum values, with percentages of 21800%, 20800%, and 1442.68%, respectively. The RMS shows the minimum response to changes in the state of the angle *θ*, with a percentage of 1.97%. When the braking time level is level 2, PPV and PV reach their maximum values, with percentages of 49.2%. As the braking time changes, the frequency domain indicators RVF, RMSF, and FC have high sensitivity to changes in the healthy state of the angle *θ* in Fig 7(b). As the braking time level continues to increase, the indicator FC of the angle *θ* decreases. RVF and RMSF decrease first and then increase. FC achieves the minimum value of 0.65% in level 4 braking time. The RVF and RMSF reach their minimum value in level 2 braking time, with a percentage of 0.017%.

**Fig 8:**
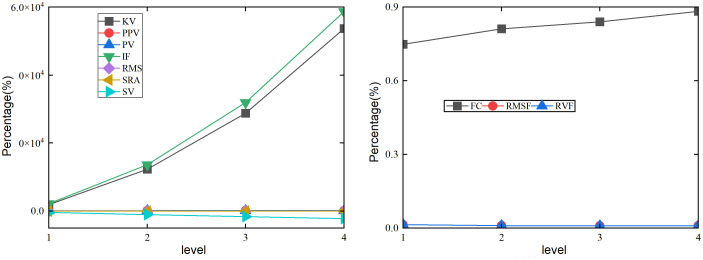
Effect of the different braking time on angle under the lifting and slewing coupling motion. (a) Time domain indicators of *β*. (b) Frequency domain indicators of *β*.

As the braking time changes, the sensitivity of the time-domain indicator SRA is poor in changes in the healthy state of the swing angle *β* in Fig 8(a). The sensitivity of the time-domain indicators KV, PPV, IF, RMS, PV, and SV are high to changes in the healthy state of the angle *β*. As the braking time level increases, the health status changes of KV, IF, RMS, and SV on the suspension swing angle show monotonicity. PPV and PV have a consistent trend in the health status changes of angle *β*. When the braking time level is 4, the response of IF and KV to the state change of the suspension swing angle show the maximum values, with percentages of 58700% and 53700%, respectively. The response of SV and RMS to changes in the state of suspension swing angle show the minimum values, with percentages of −2180.48% and 4.45%, respectively. When the braking time level is 3, PPV and PV reach their maximum values, with percentages of 156%. As the braking time changes, the frequency domain indicator FC has a high sensitivity to changes in the healthy state of the angle *β* in Fig 8(b). The frequency domain indicators RVF and RMSF have good stability in changing the health status of the angle *β*. As the braking time level continues to increase, the indicator FC continues to increase. FC achieves its maximum value in level 4 braking time, with a percentage of 0.88%.

**Fig 9:**
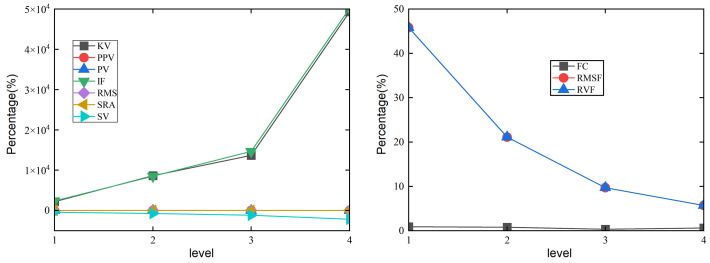
Effect of the different braking time on plate under the lifting and slewing coupling motion. (a) Time domain indicators of *w*. (b) Frequency domain indicators of *w*.

When the braking time of the coupling system is changed continuously under the lifting and slewing motion, the time-domain indicators SRA, PPV, RMS, and PV have higher stability in changing the status of the foundation plate displacement in Fig 9(a). The time-domain indicators KV, IF, and SV have higher sensitivity in changing the health status of the foundation plate. As the braking time level increases, the changes of KV, IF, and SV on the displacement of the foundation plate exhibit monotonicity. After braking time level reaches level 3, the rate of change in the displacement percentage of the foundation plate significantly increases. When the braking time level is 4, the state change response of IF and KV show the maximum values, with percentages of 50200% and 49300%, respectively. The state change response of SV shows the minimum value, with percentages of −2149.81%. As the braking time changes in Fig 9(b), the RVF and RMSF have high sensitivity to changes in the healthy state of the foundation plate. The FC has good stability in changing the health status of the foundation plate. As the braking time level continues to increase, the RVF and RMSF of the displacement percentage of the foundation plate continue to decrease. RVF and RMSF achieve the minimum value of 5.69% in the 4th level braking time.

**Fig 10:**
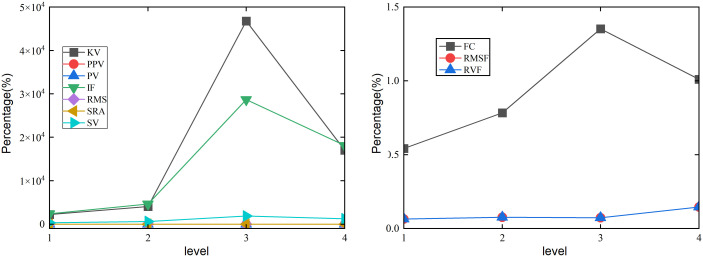
Effect of the various braking time on angle under the luffing and slewing coupling motion. (a) Time domain indicators of *θ*. (b) Frequency domain indicators of *θ*.

As the braking time of the system decreases under the luffing and slewing motion in Fig 10(a), the sensitivity of the SRA is poor to changes in the healthy state of the swing angle *θ*. The sensitivity of the KV, PPV, IF, RMS, PV, and SV are high in changes of the healthy state of the angle *θ*. As the braking time level increases, the trend of changes in KV, IF, and SV first increases and then decreases. The RMS shows a monotonic decrease. The health trend of PPV and PV on the angle *θ* first decreases, then increases, and then decreases. When the braking time level is not less than level 2, the indicators KV, PPV, IF, PV, and SV show significant fluctuations. When the braking time level is 3, the response of KV, PPV, IF, PV, and SV to the state change of the angle *θ* show the maximum values, with percentages of 46800%, 6.55%, 28700%, 6.55%, and 1850.24%, respectively. When the braking time level is 4, the RMS shows the minimum response, with a percentage of 0.06%. As the braking time changes in Fig 10(b), the FC has a high sensitivity to changes in the healthy state of the angle *θ*. The sensitivity of the RVF and RMSF to changes in the health status is slightly lower. As the braking time level continues to increase, the indicator FC first increases and then decreases. The indicator FC achieves its maximum value in level 3 braking time, with a percentage of 1.35%.

**Fig 11:**
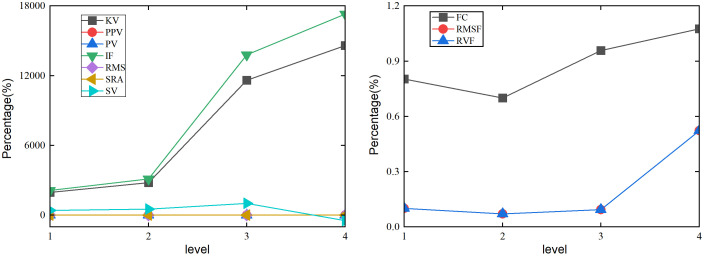
Effect of the various braking time on the angle under the luffing and slewing coupling motion. (a) Time domain indicators of *β*. (b) Frequency domain indicators of *β*.

As the braking time changes in Fig 11(a), the sensitivity of the SRA and RMS to changes is poor in the healthy state of the swing angle *β*. The sensitivity of the KV, PPV, IF, PV, and SV to changes is high. As the braking time level increases, the health status changes of KV and IF show monotonicity. The trend of changes in PPV, PV, and SV first increase and then decrease. When the braking time level is not less than level 2, the variation trend of IF and KV fluctuate greatly. When the braking time level is 4, the response of IF and KV show the maximum values, with percentages of 17300% and 14600%, respectively. When the braking time level is level 3, the SV shows the maximum response to the state change of the angle *β*, with a percentage of 1013.37%. When the braking time level is level 2, PPV and PV reach their maximum values, with percentages of 2.29%. As the braking time changes in Fig 11(b), the FC, RVF, and RMSF have high sensitivity to changes in the healthy state of the swing angle *β*. As the braking time level continues to increase, the FC, RVF, and RMSF first decrease and then increase. Their minimum value appears at level 2 of braking time, with percentages of 0.7%, 0.07%, and 0.07%, respectively. Their maximum braking time is achieved at level 4, with percentages of 1.07%, 0.52%, and 0.52%, respectively.

**Fig 12:**
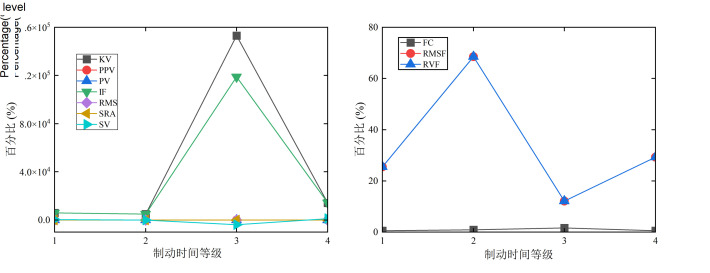
Effect of the various braking time under the luffing and slewing coupling motion. (a) Time domain indicators of *w*. (b) Frequency domain indicators of *w*.

The braking time of the system constantly changes under the luffing and slewing coupling motion in Fig 12(a), and the sensitivity of the SRA, PPV, RMS, and PV to is poor in the healthy state change of the foundation plate. The sensitivity of the KV, IF, and SV to is high. As the braking time level increases, the health status changes of KV and IF first decrease, then increase, and finally decrease. Their maximum percentages at level 3 are 153000% and 119000%, respectively. Their minimum percentages of KV and IF at level 2 are 4996.19% and 5003.17%, respectively. The trend of SV changes first decreases and then increases, with the maximum and minimum percentages being 1155.73% and −3901.47%, respectively. As the braking time changes in Fig 12(b), the RVF and RMSF have high sensitivity to changes of the foundation plate. The FC has slightly better stability in changing the status of the foundation plate. As the braking time level continues to increase, the RVF and RMSF first increase, then decrease, and then increase. The RVF and RMSF achieves the minimum value of braking time at level 3, with percentages of 12.12% for both. They achieve their maximum value at level 2, with percentages of 68.50%.

## Conclusions

The dynamic response and indicator analysis of the tower crane and foundation plate system model is investigated under dynamic and complex excitation. A test bench was designed for verification purposes. The impacts of braking time and coupling motion on the structural vibration of the system are deliberated.

An increment in the emergency braking time results in an augmentation of the amplitude of the foundation plate and angle *θ* under the lifting and slewing coupling motion. When the emergency braking time does not exceed 1.5 seconds, the structural vibration of the tower crane and foundation plate system can fulfill the relevant safety criteria. The KV, IF, and SV exhibit high sensitivity to the alterations in the status of the swing angle and foundation plate under the lifting and slewing coupling motion. The FC demonstrates high sensitivity to the changes in the status of the swing angle. The RVF and RMSF display high sensitivity to the changes in the status of the foundation plate.

As the emergency braking time diminishes under the luffing and slewing coupling motion, the structural vibration of the tower crane and foundation plate system becomes less pronounced. When the emergency braking time is 1.5 seconds, the displacement of the foundation plate and the structural vibration of the tower crane are conspicuous. The indicators KV, IF, and SV exhibit a high degree of responsiveness to the changes in the health condition of the foundation plate and the swing angle. The frequency domain indicator FC is highly sensitive to the status change of the swing angle. The frequency domain indicators RVF and RMSF are highly sensitive to the status change of the plate.
